# Radial nerve palsy caused by a rapidly growing intramuscular hematoma in an infant with biliary atresia: a case report

**DOI:** 10.1186/s12887-023-04071-5

**Published:** 2023-05-19

**Authors:** Kohei Kawahara, Koki Ota, Shingo Numoto, Nami Nakamura, Ryosuke Miyamoto, Hitoshi Honma, Yusuke Morishita, Katsuhisa Kawanami, Nozomi Matsushita, Shoko Kato, Kenitiro Kaneko, Akihisa Okumura, Hideyuki Iwayama

**Affiliations:** 1 Department of Pediatrics, Aichi Medical University School of Medicine, 1-1, Yazakokarimata, Nagakute, Aichi 480-1195 Japan; 2 Postgraduate Clinical Training Center, Aichi Medical University School of Medicine, Nagakute, Aichi Japan; 3 Department of Orthopedics, Aichi Medical University School of Medicine, Nagakute, Aichi Japan; 4 Department of Surgery, Aichi Medical University School of Medicine, Nagakute, Aichi Japan

**Keywords:** Biliary atresia, Vitamin K deficiency bleeding, Intramuscular haematoma, Radial nerve palsy, Wrist drop

## Abstract

**Background:**

Biliary atresia (BA) is a rare cause of persistent jaundice in infants that can result in vitamin K malabsorption and vitamin K deficiency bleeding (VKDB). We present an infant with BA who developed a rapidly growing intramuscular hematoma in her upper arm after a vaccination which caused a radial nerve palsy.

**Case presentation:**

An 82-day-old girl was referred to our hospital because of a rapidly growing left upper arm mass. She had received three doses of oral vitamin K before age 1 month. At age 66 days, she received a pneumococcal vaccination in her left upper arm. On presentation, she showed no left wrist or finger extension. Blood examination revealed direct hyperbilirubinemia, liver dysfunction, and coagulation abnormalities, indicating obstructive jaundice. Magnetic resonance imaging showed a hematoma in the left triceps brachii. Abdominal ultrasonography revealed an atrophic gallbladder and the triangular cord sign anterior to the portal vein bifurcation. BA was confirmed on cholangiography. VKDB resulting from BA in conjunction with vaccination in the left upper arm were considered the cause of the hematoma. The hematoma was considered the cause of her radial nerve palsy. Although she underwent Kasai hepatic portoenterostomy at age 82 days, the obstructive jaundice did not sufficiently improve. She then underwent living-related liver transplantation at age 8 months. The wrist drop was still present at age 1 year despite hematoma resolution.

**Conclusions:**

Delayed detection of BA and inadequate prevention of VKDB can result in permanent peripheral neuropathy.

## Background

Biliary atresia (BA) is an idiopathic disease of the bile ducts which causes biliary obstruction and presents with persistent jaundice in the neonatal period and early infancy. The incidence of BA is approximately 1 in 15,000 live births in Western Europe and North America and up to 1 in 6,000 to 9,000 births in East Asia [1]. Poor bile secretion in BA patients can cause vitamin K deficiency due to malabsorption of fat and fat-soluble vitamins. Vitamin K deficiency bleeding (VKDB) may result [2], which is a potentially serious complication of BA. A previous report has indicated that BA patients can develop VKDB despite daily or weekly oral vitamin K supplementation [3]. Early detection of BA and vitamin K supplementation are important to prevent VKDB.

VKDB in infancy is classified according to the time of onset: early (within 24 h of birth), classic (within 1 week of birth), and late (between age 2 weeks and 6 months) [2]. In a Japanese national survey [4], nine of 71 cases of late VKDB were caused by BA. Intracranial hemorrhage is the most common hemorrhagic complication in patients with late VKDB, followed by gastrointestinal hemorrhage and intramuscular hematoma [4].

An intramuscular hematoma in the upper arm may cause damage or compression of the radial nerve, resulting in radial nerve palsy [5], which presents as wrist drop or an inability to fully extend the wrist and fingers. Common causes of radial nerve palsy in an infant include trauma and nerve compression or entrapment [6]. Radial nerve palsy can be temporary or permanent, depending on the underlying cause and extent of nerve damage. To the best of our knowledge, radial nerve palsy caused by intramuscular hemorrhage in the upper arm has not been previously reported. We report such a case in an infant with BA.

## Case presentation

**Fig. 1:**
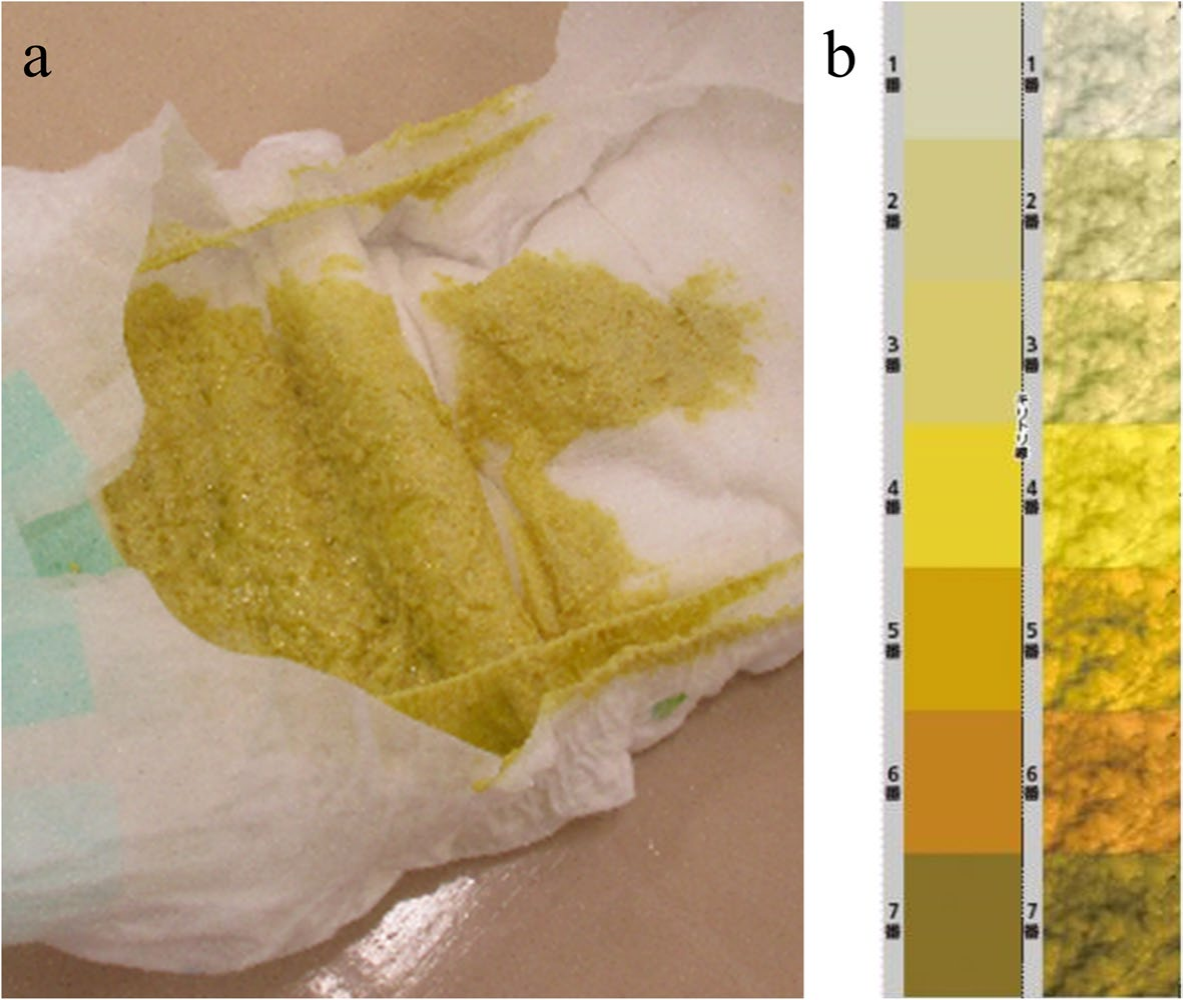
**a** The patient’s stool. **b** The stool color card used in Japan

An 82-day-old girl was referred to our hospital because of a rapidly growing mass in her left upper arm. She was born via an uncomplicated vaginal delivery at 37 weeks of gestation and had a birth weight of 2700 g. She received three 2 mg doses of oral vitamin K (at birth, age 5 days, and age 1 month). At 1 month of age, her stool color was number 4 on the stool color card (Fig. 1). She was fed a combination of formula and breast milk. Initial weight gain was 33 g/day, which subsequently decreased to 14 g/day. At age 66 days during a routine vaccination, her mother expressed concern about persistent jaundice, which was attributed to breastfeeding jaundice. The infant received a pneumococcal vaccination in her left upper arm and those for *Haemophilus influenzae* type b, hepatitis B, diphtheria-pertussis-tetanus, and polio in other extremities. No other episodes of left upper arm trauma were reported.

**Fig. 2:**
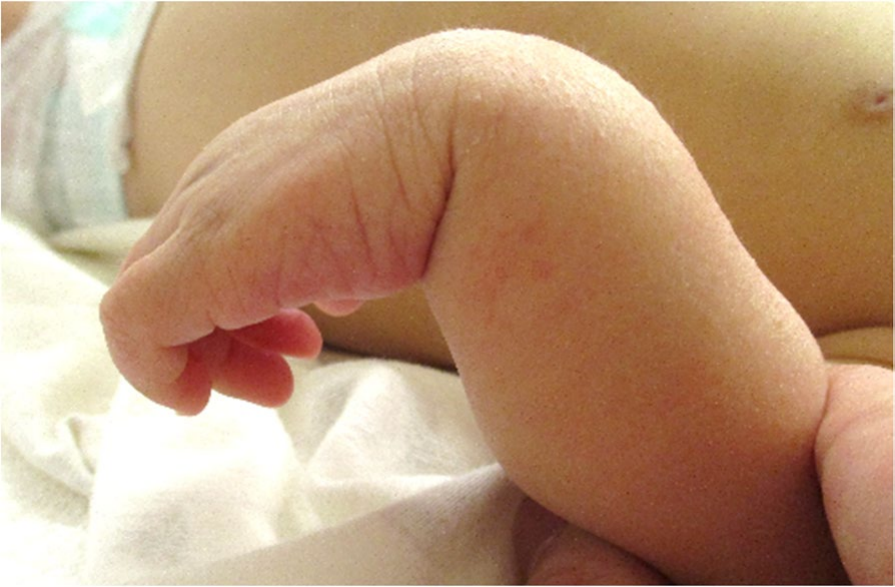
The patient had a left wrist drop, as she demonstrated no extension at the left wrist or metacarpophalangeal joints

On presentation, the patient was alert with generalized jaundice and hepatomegaly. A 6 cm × 4 cm × 4 cm hard elastic mass was found on the medial side of the left upper arm, which was noticed by her mother the previous night. She was not extending her left wrist or fingers (Fig. 2). Blood testing revealed direct hyperbilirubinemia (total bilirubin [TBil], 14.2 mg/dL; direct bilirubin [DBil], 10.0 mg/dL; bile acid, 109.1 µmol/L) and liver dysfunction (aspartate aminotransferase [AST], 273 U/L; alanine aminotransferase [ALT], 233 U/L; alkaline phosphatase [ALP], 3306 U/L; glutamyl transpeptidase [GTP], 1551 U/L), indicating obstructive jaundice. Prothrombin time (PT) and activated partial thromboplastin time (APTT) were < 10% (normal range, 85–100%) and > 180 s (reference, 26.7 s), respectively (Table 1). Concentrations of protein induced by vitamin K absence-II, vitamin K, and other fat-soluble vitamins were not measured because of a lack of blood samples [7].

**Table 1:** Laboratory data initially and after parenteral administration of vitamin K and transfusion of fresh frozen plasma

Laboratory data	Reference range	On admission	After treatment with parenteral vitamin K and fresh frozen plasma
TBil (mg/dL)	4.2–8.8	14.2	19.6
DBil (mg/dL)	< 2	10.0	14.1
Bile acid (µmol/L)	0–8	109.1	49.0
AST (U/L)	32.9–40.7 U/L	273	322
ALT (U/L)	11.8–15.4 U/L	233	256
ALP (U/L)	210–510 U/L	3306	3541
GTP (U/L)	0–20 U/L	1551	1499
PT (%)	85–100	< 10	99
APTT (second)	reference: 26.7	> 180	34.9

*ALP* Alkaline phosphatase, *ALT* Alanine aminotransferase, *APTT* Activated partial thromboplastin time, *AST* Aspartate aminotransferase, *DBil* Direct bilirubin, *GTP* Glutamyl transpeptidase, *PT* Prothrombin time, *TBil* Total bilirubin

**Fig. 3:**
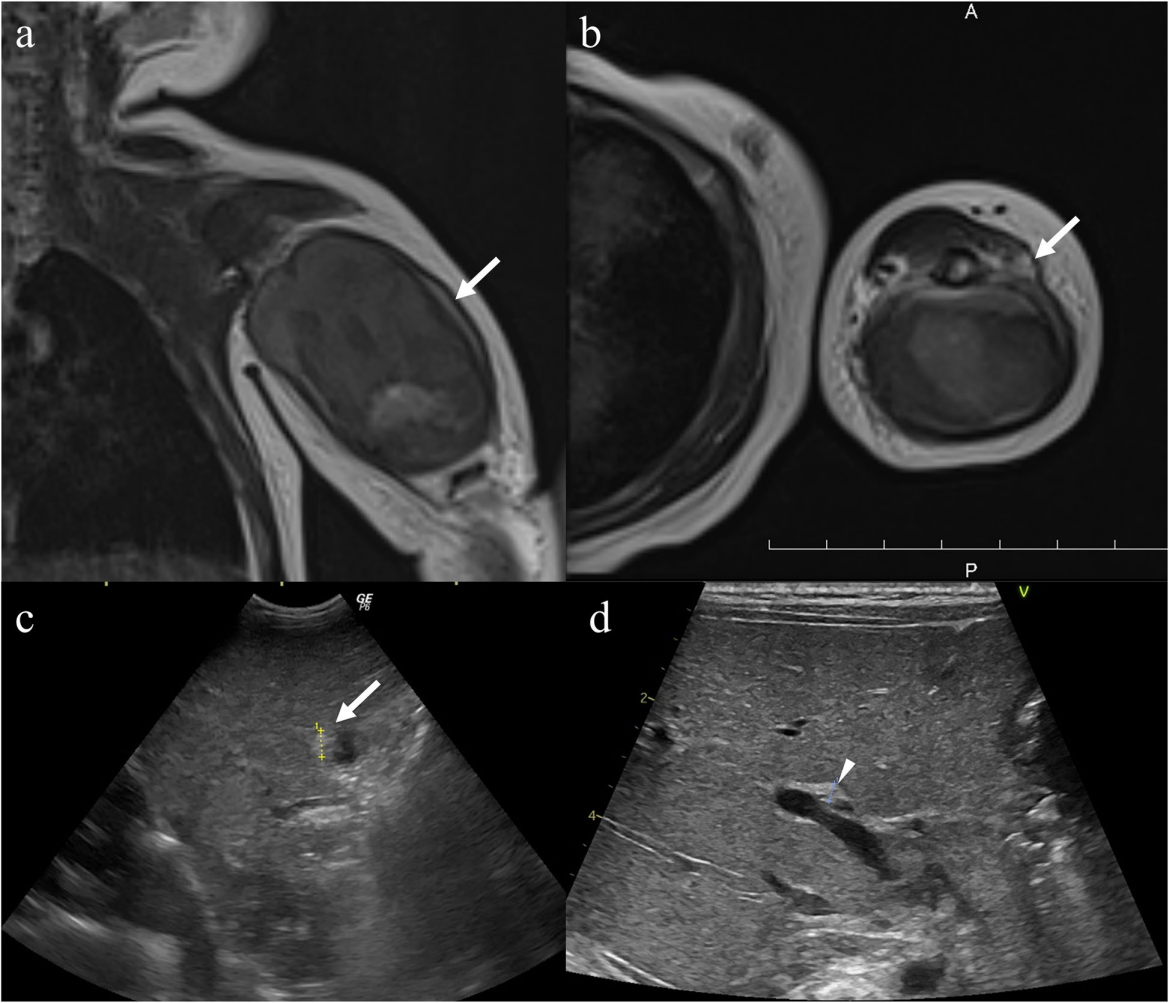
Coronal (**a**) and axial (**b**) magnetic resonance images of the left upper arm. The arrows indicate a hematoma in the triceps brachii. No changes in signal intensity were observed in the subcutaneous tissue. The radial nerve was not clearly visualized. **c** and **d** Ultrasonographic images of the abdomen. The arrow in **c** indicates the gallbladder (diameter, 7.5 mm). The arrow head in **d** indicates the triangular cord sign anterior to the bifurcation of the portal vein

Magnetic resonance imaging revealed a hematoma in the left triceps brachii (Fig. 3a), which we presumed was compressing the radial nerve (Fig. 3b). No humeral fracture was found. Abdominal ultrasonography showed an atrophic gallbladder (Fig. 3c) and the triangular cord sign anterior to the portal vein bifurcation (Fig. 3d). The choledochal duct was not visible on magnetic resonance cholangiopancreatography. Head computed tomography revealed no intracranial hemorrhage.

Intramuscular hematoma caused by BA-related VKDB was diagnosed and the infant received parenteral vitamin K (2 mg daily) and a transfusion of fresh frozen plasma. Her coagulation abnormalities improved after 5 days (PT, 99%; APTT, 34.9 s; Table 1) and the hematoma gradually decreased in size.

Cholangiography was performed to confirm the diagnosis of BA, which showed no passage of contrast into either the intra- or extrahepatic biliary systems. She then underwent Kasai hepatic portoenterostomy at age 82 days. However, her jaundice did not sufficiently improve (TBil, 8.9 mg/dL; DBil, 6.2 mg/dL). Therefore, she underwent living-related liver transplantation from her father at age 8 months. After transplantation, her liver function improved significantly (TBil, 0.9 mg/dL; DBil, 0.03 mg/dL; bile acid, 14.9 µmol/L; AST, 45 U/L; ALT, 28 U/L; ALP, 523 U/L; and GTP, 14 U/L). However, the wrist drop was still present at age 1 year.

## Discussion and conclusions

The patient presented here developed a rapidly expanding intramuscular hematoma and consequent radial nerve palsy owing to VKDB that resulted from BA. Three doses of oral vitamin K in her first month of life failed to prevent VKDB. These facts suggest that delayed recognition of BA and inadequate prevention of VKDB may have permanent neurological consequences.

Vaccination in the setting of VKDB is the probable cause of our patient’s intramuscular hematoma. In a report from Indonesia, 32 of 55 cases of post-vaccination hemorrhage were caused by VKDB [8]. A recent study in Nepal reported 16 cases of hemorrhage in 15,350,165 injectable vaccine doses; 14 of these were caused by VKDB [9]. These data suggest that VKDB should be considered a major risk factor for bleeding after vaccination. Our patient received a routine vaccination 2 weeks before an intramuscular hematoma developed at the site. However, her mother reported that it developed rapidly over a short period of time. If the vaccination induced the hematoma, it may have grown slowly. Therefore, the vaccination may have triggered hematoma development and VKDB promoted rapid expansion.

Vitamin K supplementation is necessary to prevent VKDB. In Japan, oral vitamin K is routinely administered to newborn infants three times (at birth, age 5 to 7 days, and age 1 month); however, this does not completely prevent VKDB [10]. Intramuscular vitamin K administration can prevent VKDB, intracranial hemorrhage, and multiple hemorrhages in infants with BA [11]. Thirteen weekly doses of oral vitamin K is also widely used in newborn infants in Japan; however, this may also be insufficient to prevent VKDB in high-risk infants, such as those with BA. Establishment of a more effective means of preventing VKDB is needed in Japan.

Early detection of BA is crucial for preventing the associated VKDB [12]. However, a suitable screening test is lacking. In a meta-analysis of population-based BA screening methods, the stool color chart had a low sensitivity for detection; in contrast, TBil and DBil concentrations had both high sensitivity and specificity [1]. In our patient, the mother expressed concern about persistent jaundice during routine vaccination, but the attending physician at the time diagnosed it as breastfeeding jaundice. If TBil and DBil had been measured, BA would probably have been detected. Measurement of both should be considered in infants with persistent jaundice [1].

In conclusion, VKDB appears to be a risk factor for post-vaccination intramuscular hematoma. Vitamin K supplementation and early detection of BA are crucial to prevent VKDB in infants with BA. Delayed detection of BA and inadequate prevention of VKDB can result in permanent peripheral neuropathy.

## Data Availability

The data that support the findings of this study are available upon request from the corresponding author. The data are not publicly available because of privacy and ethical restrictions.

## Abbreviations

ALP: Alkaline phosphatase
ALT: Alanine aminotransferase
APTT: Activated partial thromboplastin time
AST: Aspartate aminotransferase
BA: Biliary atresia
DBil: Direct bilirubin
GTP: Glutamyl transpeptidase
PT: prothrombin time
TBil: total bilirubin
VKDB: vitamin K deficiency bleeding
